# A Delphi consensus report of the Italian society of thoracic surgery on influencing factors, use of a bleeding scale, and management of bleeding in thoracic surgery

**DOI:** 10.3389/fsurg.2026.1734148

**Published:** 2026-04-28

**Authors:** Domenico Galetta, Federico Rea, Stefano Margaritora, Agostini Vanessa, Francesco Facciolo, Franca Melfi, Gianluca Guggino, Roberto Crisci, Collaborators and Participating Institutions

**Affiliations:** 1 Division of Thoracic Surgery, San Giovanni Bosco Hospital, ASL Città di Torino, Turin, Italy; 2 Unit of Thoracic Surgery, Department of Cardiac, Thoracic, Vascular Sciences and Public Health, Padua University Hospital, Padua, Italy; 3 Department of Thoracic Surgery, Università Cattolica del Sacro Cuore, IRCSS Fondazione Policlinico Universitario Agostino Gemelli, Rome, Italy; 4 Transfusion Medicine Department, IRCCS-Ospedale Policlinico San Martino, Genoa, Italy; 5 Thoracic Surgery Unit, IRCCR Regina Elena National Cancer Institute, Rome, Italy; 6 Surgical, Robotic Center, Italy and University of Calabria, Pisa, Italy; 7 Thoracic Surgery Unit, Antonio Cardarelli Hospital, Naples, Italy; 8 Thoracic Surgery, University of L’Aquila, L’Aquila, Italy

**Keywords:** bleeding, thoracic surgery, hemostasis, coagulation, Delphi consensus

## Abstract

**Introduction:**

We report the results of a national consensus paper among Italian thoracic surgeons obtained through a Delphi process, on the bleeding in thoracic surgery (BTS) evaluating influencing factors, use of a validated intraoperative bleeding scale (VIBeS), its management and helping improving practice.

**Methods:**

A panel of 20 statements (a total of 39 issues) was developed and, after initial validation by six experts, was electronically sent to 60 Italian thoracic surgeons. Participants were asked to score each statement on a 5-point Likert scale and the agreement was scored for evaluating the consensus (>66%).

**Results:**

Overall, a total of 49 (82%) participants scored the proposed statements. The consensus was reached in 35/39 issues (89.7%). Responders agreed (>90%) that medical (comorbidities, anticoagulant or antiplatelet therapies) and surgical factors (pleural adhesions, procedures on parietal pleura and chest wall resection) influenced BTS. Use of VIBeS has gained broad positive acceptance (>90%). Effects of BTS have achieved a broad positive consensus both for intraoperative and post-operative ones (surgeon stress, overall costs, length of operation, postoperative complications) (>73%). Modality of reduction and management of BTS (use of appropriate hemostatic products according to the coagulation status and VIBeS) has obtained a broad positive consensus.

**Conclusions:**

The expert panel of Italian thoracic surgeons reached an agreement on the majority of issues of Delphi survey (factors, effects and management of BTS). The use of a VIBeS is recommended. In the absence of prospective and randomized studies on management of BTS, this document may be useful for reducing practice variation among thoracic surgeons facing intraoperative bleeding.

## Introduction

Bleeding is one of the main complications of thoracic surgery and can occur both intraoperatively or postoperatively. While the occurrence is rare, it can be life threatening (1, 2). Causes of intraoperative bleeding in thoracic surgery may be more frequently related to the presence of pleural adhesions whose dissection may be the most common site of hemorrhage, to the surgical procedure (lung resection, lymph node dissection), to a damage of pulmonary vessels during the isolation and resection.

Different strategies may be adopted for the control of the intraoperative bleeding depending from the entity of the hemorrhage: in most cases, temporary electrocoagulation hemostasis may be enough to stop the bleeding. In case of major vascular bleeding by a venous or arterial vessel, when the simple and prolonged compression with a gauze is not sufficient to stop bleeding, it may be necessary to control the vessel and repair it by applying clips or suturing or ligating the vessels.

Postoperative bleeding is a rare complication after pulmonary resection (0.1%–3%) (3–9); nevertheless, it may be responsible of hemodynamic instability and require aggressive treatment. The incidence of reoperation due to postoperative hemorrhage after lung resection is approximately 2.6% (4). It represents the most common indication for reoperation after lung resection, accounting for approximately 26.6%–73.3% of cases (10, 11). It may be more frequently related to the shedding of the eschar of an intraoperatively controlled hemorrhage caused by the reconstitution of postoperative intrapleural negative pressure. Other bleeding sites of reoperation, such us pulmonary artery branch, pulmonary artery trunk, intercostal blood vessel and bronchial artery were mainly considered to result from technical issues during surgical procedures, including insecure vessel ligation, simple hemostasis by compression, or single hemostasis performed by electrocoagulation.

Alongside surgical problems, there are also preoperative risk factors that increase the likelihood of bleeding in thoracic surgery. At least 25% of patients undergoing any surgery are taking some form of prescribed anticoagulation including various antiplatelet agents, including aspirin (12), for cardiovascular diseases (deep venous thrombosis, pathologic hypercoagulopathy, atrial fibrillation, or cerebrovascular disease). These drugs should be accordingly discontinued preoperatively and the routine coagulation screen together with an assessment of full blood count, ferritin, transferrin receptor should be evaluated before surgery in order to reduce the risk of bleeding. Other comorbidities may predispose a patient to bleeding and include preoperative anemia, neoadjuvant therapy (13), and vascular disease which can lead both to physical impairment and to increased vulnerability of blood vessels.

Surgical hemostats and tissue sealants are adjuncts whose function is to consolidate and complement surgical techniques for hemostasis. Hemostatic agents are topical and absorbable adjuncts which are used on the surgical bleeding sites for consolidate the local hemostasis (14). There are a number of topical hemostatic agents currently available for use in surgery which can be broadly divided, according to their mechanism of action, into passive or active, along with tissue sealants (15) (Table 1). While passive hemostats are dependent on a patient's own clotting cascade to achieve durable hemostasis, active hemostats are effective in patients with deranged coagulation by providing extraneous fibrinogen and/or thrombin, thus playing a proactive role in clot generation. The mechanism of action of passive hemostatic agents occurs through (a) the passive contact activation and (b) by providing a support structure to platelet aggregation and clot formation. Thus, Passive the hemostatic agents with non-flowable features are often used to act on the bleeding ranging from mild capillary oozing to more broad moderate bleeding. Because of the mechanism of action of passive hemostats is dependent on a functioning coagulation cascade, in patients with coagulation disorders or those treated with antiplatelet medications or anticoagulant drugs, the efficacy of passive agents is reduced. Other hemostatic products as fibrin sealants, advanced patches, or active flowable hemostatic agents (i.e., thrombin with gelatin), promote hemostasis directly by acting at the end of the coagulation cascade accelerating the formation process of the natural clot. They are effective regardless of whether patients have been treated with anticoagulant or antiplatelet medications, and function independently of the patient's ability to generate clotting factors. Finally, there are topical sealants which may be synthetic (cyanoacrylates or polyethylene glycol), semi-synthetic (glutaraldehyde–albumin), or fibrin-based.

**Table 1:** Indications and types of surgical haemostats and tissue sealants.

Type of surgical haemostats and tissue sealants	Types	Mode of action	Bleeding range	Patient factors
Passive	Oxidised cellulose (regenerated)	Contact activation and platelet aggregation	Mild (capillary oozing) to moderate bleeding	Intact coagulation
				Limited effect in heparinised and/or anticoagulated treated patients
	Collagens			
	Powders	Relies on the patient's ability to generate clotting factors		
	Gelatin sponges			
	Polysaccharide spheres			
Active	Flowables (thrombin and gelatin)	Functions independently of the patient's ability to generate clotting factors	Broad range of active bleeding	Compromised coagulation
	Fibrin sealants			Effective in heparinised and/or anticoagulated treated patients
	Advanced patches (with fibrin sealants or polyethylene glycol)			
Tissue sealants	Fibrin based	Functions independently of the patient's ability to generate clotting factors	Pre-emptive for anticipated bleeding	Compromised coagulation
	Synthetic (i.e., cyanoacrylates or polyethylene glycol)			
				Effective in heparinised and/or anticoagulated treated patients
	Semi-synthetic (i.e., glutaraldehyde–albumin)			

**Figure 1:**
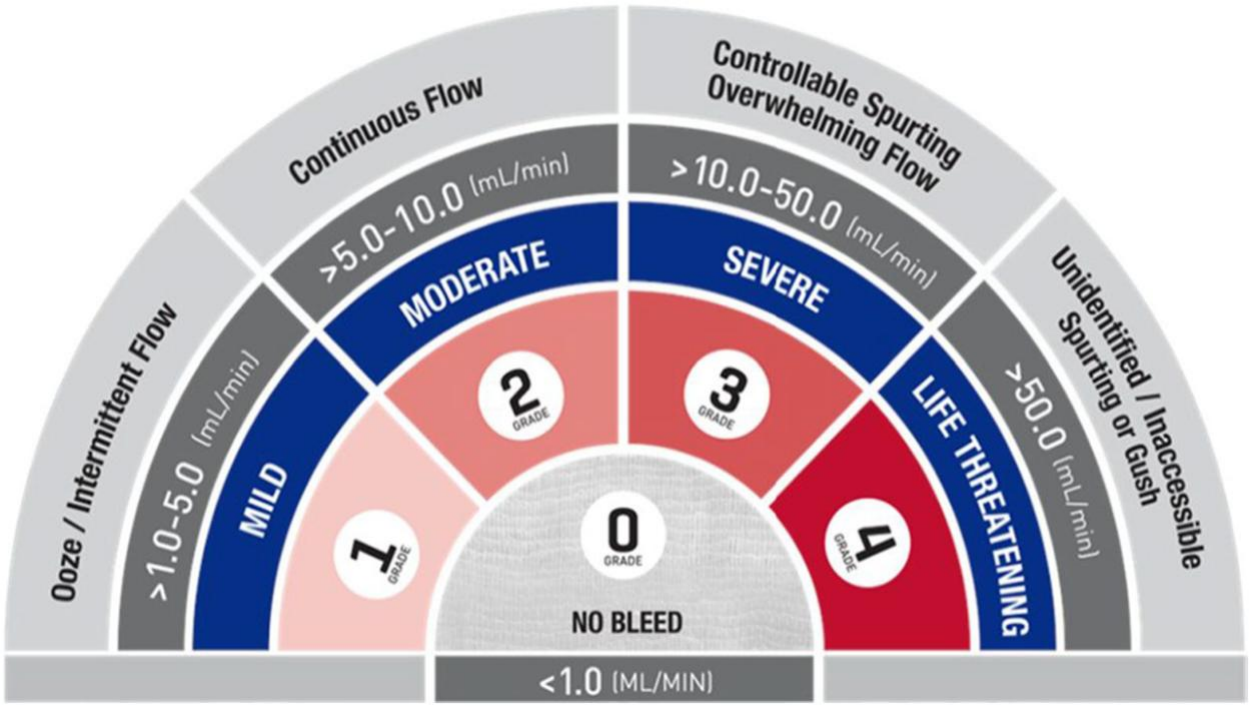
Validated intraoperative bleeding scale (VIBeS) grading. VIBeS grades are based on visual field and anatomic appearance, qualitative description, and visual estimated blood loss. Grade 0: No bleeding; reflects clinically insignificant and unremarkable bleeds. Grade 1: Mild; bleeds represent a general ooze, which well up over 1–2 min after blotting with gauze. Grade 2: Moderate; bleeds visibly well up after blotting, and are usually considered distracting to the surgical procedure. Grade 3: Severe; bleeds well up immediately after blotting, and require treatment to continue with the surgery. Grade 4: Life threatening; Grade 4 bleeds are life-threatening and require immediate surgical treatment (17).

Recently, an interesting tool which may facilitate the accurate and reproducible description of bleeding during surgery has been reported: it is the validated intraoperative bleeding scale (VIBeS) which has been developed and validated amongst surgeons from different surgical specialties (Figure 1) (16, 17). The VIBeS includes five different points which help the surgeon to grade intra-operative bleeding according to a different severity grade of bleeding based on the visual and anatomical appearances considering also the qualitative description, and the estimated rate of blood loss. Initially used in clinical trials for comparing the efficacy of different hemostatic agents, the VIBeS has been adopted for evaluating the intra-operative surgical bleeding because its ability for a best intraoperative assessment of bleeding. In fact, the VIBeS introduces a common language to describe bleeding for surgeons, theatre scrub staff, and anesthetists allowing all the involved personnel to better manage the intraoperative bleeding event and, for the surgeon, to choice of the most appropriate hemostatic agent for the grade of bleeding for rapid and consistent hemostasis (18). Other advantages in the use of the VIBeS are the following: it allows to less waste and more effective use of hemostatic adjuncts, to reduce peri-operative blood loss and need for blood and blood product transfusion, the potential for overall improved patient outcomes, and economic impact allowing the selection of the optimal and adequate hemostatic agent (19).

The aim of this study is to report the results of a national consensus paper among Italian thoracic surgeons obtained through a Delphi process, on the bleeding in thoracic surgery (BTS) evaluating influencing factors, use of a VIBeS, its management and helping improving practice.

## Methods

Delphi methodology is a consensus-based technique that systematically collects and aggregates opinions from a group of experts within a medical specialty via administration of questionnaires (20, 21). Several studies have demonstrated the value of the Delphi method in areas of health care and epidemiology, mainly when robust forms of evidences such as randomized controlled trials were unavailable (22). The ideal number of participants required to obtain a consensus in the medical field using the Delphi method is unknown (23). Therefore, the number of experts selected was based on prior experiences in which the Delphi method was used and on the expected response rate (22, 24).

We conducted a modified Delphi study on BTS to establish consensus on the causes, the use of a VIBeS, its management with the aim of helping improving practice.

The Delphi consensus finding process consisted of an online starting meeting and a virtual advisory board meeting using Zoom (Zoom Video Communications Inc., San Jose, California, USA) to evaluate the scientific literature on BTS, to discuss real-life clinical cases by the board of experts, hereafter referred to as key opinion leaders (KOLs), who were selected based on their previous experience in thoracic surgery, and to initiate an open discussion to suggest items needed for constructing the online Delphi questionnaire. Eight Italian experts formed the KOLs: seven thoracic surgeons (DG, FR, SM, FF, FM, GG, and RC) and one hematologist (AV). All the eight experts who planned the questionnaire signed an informed consent for participating to this study group. Although not systematic, the KOLs believed that the literature review performed included the main available evidence and represented the basis for the identification of different issues by the KOLs aiming to evaluate the factors determining BTS and its management. There were 20 statements, including a total of 39 issues, grouped into the following 4 broader categories: (i) factors influencing bleeding in thoracic surgery, (ii) use of VIBeS, (iii) effects of bleeding in thoracic surgery, and (iv) modality of reduction and management of bleeding in thoracic surgery (Table 2). Once developed, the questionnaire was distributed online to Italian thoracic surgeons (Delphi round) who were asked to rate their agreement or not to each statement, using a 5-point Likert scale where 1 = “strongly disagree” and 5 = “strongly agree”. The answers were collected anonymously and analyzed by the KOLs. In this study, a sum of 1–2 points were considered “negative consensus” while a sum of 3–5 points was considered “positive consensus” (25) (Table 3).

**Table 2:** Results of the Delphi method survey of 49 expert Italian thoracic surgeons.

**Category #1: Factors influencing bleeding in thoracic surgery**							
	**Response (*n*** **=** **49)**					**Consensus score**	
**Statement #1—The medical factors affecting the bleeding include:**	**1**	**2**	**3**	**4**	**5**	**Disagree**	**Agree**
1.1 Comorbidities (pre-existing pathologies)	1	3	23	16	6	8%	92%
1.2 Anticoagulant therapies in progress	0	0	1	15	33	0	100%
1.3 Antiplatelet therapy (single or double)	0	2	11	16	20	4%	96%
1.4 Pre-operative anemia	9	24	14	2	0	67%	33%
**Statement #2—Surgical factors affecting intra- and post-operative bleeding include:**							
2.1 The surgical approach (open, vats, rats)	5	16	16	9	3	43%	57%
2.2 The presence of pleural adhesions	0	2	15	23	9	4%	96%
2.3 The planned intervention	2	15	17	14	1	35%	65%
2.4 Emergency intervention	2	14	21	9	3	33%	67%
2.5 The enlarged surgical excision	19	23	6	1	0	86%	14%
2.6 The extension of the neoplasm	0	7	26	16	0	14%	86%
2.7 The demolition of the chest wall	0	3	24	19	3	6%	94%
2.8 Procedures on the parietal pleura	0	3	18	26	2	6%	94%
2.9 The type of medical devices used in the dissection (electrocautery, ultrasound, radiofrequency, etc.)	0	15	17	13	4	31%	69%
2.10 Vascular/parenchymal closure techniques	0	8	22	17	2	18%	84%
**Category #2: Use of standardized bleeding scales (VIBe SCALE- Validated Intraoperative Bleeding Scale)**							
	**Response (*n*** **=** **49)**					**Consensus score**	
**Statement #3**	**1**	**2**	**3**	**4**	**5**	**Disagree**	**Agree**
The use of a bleeding scale is useful and effective in management of bleeding in thoracic surgery	0	4	20	16	9	8%	92%
**Statement #4**							
In thoracic surgery, the degrees of bleeding are defined by the VIBe SCALES	0	4	28	14	3	8%	92%
**Statement #5**							
With equal bleeding, the site of origin affects the degree and his control	0	0	13	21	14	0	100%
**Statement #6**							
The VIBe Scale is valid and applicable in thoracic surgery	0	5	25	14	5	10%	90%
**Statement #7**							
It is necessary to modify and adapt the VIBe Scale to the thoracic surgery	0	8	24	15	2	16%	84%

**Category #3: Consequences of bleeding in thoracic surgery**							
	**Response (*n*** **=** **49)**					**Consensus score**	
**Statement #8**	**1**	**2**	**3**	**4**	**5**	**Disagree**	**Agree**
The consequences of bleeding are different whether intra- or postoperative	0	4	15	15	15	8%	92%
**Statement #9—The consequences of intraoperative bleeding can be:**							
9.1 Surgeon stress	1	6	19	19	4	14%	86%
9.2 Higher cost to bear	1	10	15	15	8	22%	78%
9.3 Longer duration of the intervention	0	5	10	21	13	10%	90%
9.4 Increase in postoperative complications	0	3	11	26	9	6%	94%
9.5 Lengthening of hospital stays	1	5	11	24	8	12%	88%
**Statement #10—The consequences of postoperative bleeding can be:**							
10.1 Surgeon stress	2	11	15	16	5	27%	73%
10.2 Higher cost to bear	0	5	13	21	10	10%	90%
10.3 Increase in complications	0	0	8	29	12	0	100%
10.4 Lengthening of hospital stays	0	2	6	28	13	4%	96%
**Statement #11**							
Post-operative bleeding is defined as blood loss of 100 mL/h for 2-3 hours	1	13	21	11	3	29%	71%
**Category #4: Methods of reduction and management of bleeding in thoracic surgery (damage control)**							
	**Response (*n*** **=** **49)**					**Consensus score**	
**Statement #12**	**1**	**2**	**3**	**4**	**5**	**Disagree**	**Agree**
The role of a hemostatic should not replace conventional surgical technique	0	3	9	17	20	6%	94%
**Statement #13**							
The use of hemostatics can be really effective in preventing postoperative blood loss	0	3	20	20	6	6%	94%
**Statement #14**							
The pre-existing coagulation status of the patient influences the choice of the hemostatic to use Active or Passive	0	7	22	16	4	14%	86%
**Statement #15**							
In a patient with an intact coagulation system in case of Vibe Scale grade 1 bleeding is recommended to use a passive hemostatic	1	11	25	10	2	24%	76%
**Statement #16**							
In a patient undergoing dual antiplatelet therapy in the event of Vibe Scale grade 2 or grade 3 bleeding, it is necessary to use blood components such as platelet concentrates	0	8	25	15	1	16%	84%
**Statement #17**							
There is good scientific evidence to support the use of active hemostats in patients with compromised coagulation	0	8	24	16	1	16%	84%
**Statement #18**							
The costs of the hemostatic should be included in the budget deriving from the direct and indirect costs (resources, operating room time, expense for the choice of an additional adjuvant method) of a surgical procedure	0	1	25	18	5	2%	98%
**Statement #19**							
In a patient with grade 2 or grade 3 bleeding, the use of viscoelastic coagulation monitoring tools can support the choice of hemostatic therapy	0	8	22	17	2	16%	84%
**Statement #20**							
In a patient with grade 3 or grade 4 bleeding, the availability of transfusion protocols and coagulation support are helpful	0	1	10	26	12	2%	98%

**Table 3:** Likert scale and consensus definition.

Fully disagree	Disagree	Agree	More than agree	Fully agree
1	2	3	4	5

**• Sum of answers 1-2 > 66% = Negative Consensus.**

**• Sum of answers 3-4-5 > 66% = Positive Consensus.**

**• Sum of answers 1-2 or 3-4-5 < 66% = No Consensus.**

The panelists were selected based on their expertise in the topic, with the aim to equally geographically distribute the questionnaire on the national territory. An individualized email invitation that presented the questionnaire was sent to each of the experts with a link to a secure website and 2 reminder emails were sent before the closure of the round of voting. Anonymous responses to the items were tabulated into a centralized database. The experts did not have access to the opinions of the other experts during the round of voting. The results of this unique round of voting formed the basis for the current consensus. A cutoff of 66% of agreement/disagreement for each statement was necessary to define a consensus (25). No consensus was reached if a statement received <66% of concordant replies. The KOLs were provided with the results of the Delphi round, the aggregation of the responders' answers on the 5-point Likert scale, and an online consensus meeting was hosted to discuss both areas of agreement and areas without consensus. The clinical practice was considered highly recommended if 66% or more of the experts reached an agreement. There was no confidential information required for this study. Ethic committee approval was not required, given that no patient data were used in this study. Descriptive statistics were used to analyze the results. Categorical data were reported as frequency, number and percentage. Ceiling effects could not be assessed given the relatively low numbers of expert participants (26).

## Results

Of the 60 Italian thoracic surgeons invited for this survey 49 (82%) responded to the Delphi questionnaire (Table 2). There were 3 women (6%) and 46 men (94%) whose geographical distribution along the nation has been even.

All the items, except 4, reached a positive consensus, with elevate levels of agreement, as demonstrated by the presence of a consensus ≥90% in 20 issues. The minimum level of consent was 67% for statement 2.3, while the maximum level of agreement was 100% in 3 issues. For two issues the consensus was negative (1.4 and 2.5), while no consensus was reached in two cases (issue 2.1 and 2.3).

Regarding the first investigation area (factors influencing bleeding in thoracic surgery), responders agreed (>90%) that medical (comorbidities, anticoagulant or antiplatelet therapies) and surgical factors (pleural adhesions, procedures on parietal pleura, chest wall resection, type of medical devices used in the dissection, and vascular/parenchymal closure techniques) influenced BTS. Preoperative anemia (issue 1.4) and enlarged surgical excision (issue 2.5) were not considered as factors influencing BTS. No consensus was reached for considering surgical approach (issue 2.1) and the planned intervention (issue 2.3) as risk factors for BTS (Table 2).

Regarding the second investigation area (use of standardized bleeding scales), the use of VIBeS has gained broad positive acceptance (>90%). In particular, 92% of responders agreed thar VIBeS is useful and effective in management of bleeding in thoracic surgery and that the degrees of bleeding are defined by the VIBeS (92%). VIBeS is valid and applicable in thoracic surgery (90% of agreement), and it is necessary to modify and adapt it to the thoracic surgery (83% of consensus) (Table 2).

Regarding the third investigation area (consequences of bleeding in thoracic surgery), a broad positive consensus was achieved both for intraoperative and post-operative consequences (surgeon stress, overall costs, length of operation, postoperative complications, hospital stay) (>73%). A consensus rate of 71% was reached for the definition of postoperative bleeding as blood loss of 100 mL/h for 2–3 hours (Table 2).

Regarding the fourth investigation area (methods of reduction and management of BTS), a broad positive consensus was obtained about the use of appropriate hemostatic products according to the coagulation status and VIBeS (Table 2).

## Discussion

The goal of the present consensus report was to explore among Italian thoracic surgeons their attitude towards BTS evaluating influencing factors, use of a VIBeS, and the management of bleeding with the intent of improving practice. The present project involved a total of 68 expert Italian thoracic surgeons (8 KOLs and 60 selected panelists of which 48 responders) with almost 10 years of experience in the thoracic field, and evenly distributed throughout the country. There was a broad consensus about the medical factors affecting BTS (>92%) (pre-existing pathologies and anticoagulant and antiaggregant therapies). Preoperative anemia was not considered as a factor affecting BTS (borderline value of 67%). This issue was a matter of discussion among the KOLs who considered this absence of agreement by responders as the absence of perception/awareness of the impact of anemia in the patient's surgical preparation pathways. Preoperative anemia is common in thoracic surgery and may be the result of inadequate erythropoiesis due to iron deficiency, malnutrition, malabsorption, inflammation, bone marrow disorders, or chronic blood loss (27) and international recommendations strongly recommend to detect and manage anemia sufficiently early before major elective surgery (28). The KOLs accepted as definitive the result of item 1.4 considering the possibility that responders may have interpreted the presence of anemia as having more influence on postoperative hemoglobin levels than on the risk of intraoperative bleeding and taking for granted the fact of not operating on patients with hemoglobin levels below the critical threshold.

About surgical factors influencing BTS, two items did not reach the consensus (2.1 and 2.3). The failure to reach the consensus on these items was interpreted as the expression of the surgeon's learning curve given that the panel of responders was composed of surgeons of different age groups, experience and relative confidence in the surgical approaches mentioned. The negative consensus achieved with the item 2.5 was judged “anomalous” by KOLs because it contrasts with the two following items linked to the same concept of extension of the exeresis. This was interpreted as the possibility that responders may have been misled and considered the type of intervention out of context. Also, these results were accepted by KOLs as definitive without the need to perform other round of questions.

Regarding the use of a VIBeS, there was a broad agreement on its utility in thoracic surgery for the management of BTS and also for the choice of the appropriate hemostatic even if 83% of responders agreed on the necessity to modify and adapt it to thoracic surgery. In fact, as reported by some previous studies in different specialties (18, 19), the VIBeS has proven to be helpful with the advantage of a common language to describe intra-operative bleeding and to guide the most effective hemostasis.

About the consequences of BTS, a broad agreement was reached by responders both for intraoperative and postoperative consequences including surgeon stress, overall costs, length of operation, postoperative complications, hospital stay with a consensus of 73% about the definition of postoperative bleeding.

Statements related to methods of reduction and management of bleeding in thoracic surgery reached a broad consensus among responders above all about the appropriate use of hemostatic (active or passive) according to the coagulative status of the patient and the VIBeS. The types of topical hemostatic (active or passive) most used in thoracic surgery are, in order of importance, topical hemostatic, adhesive hemostatic, pure adhesives or sealants based on polyethylene glycol (PEG) or PEG polymers. Topical hemostatic agent are usually used in particular cases: a) modest bleeding/hematoma of great vessels and in oozing bleedings, b) in the hemostasis of vascular stumps and sutures, or c) in involuntary vascular lesions in “sensitive” areas (e.g., close to the esophagus or phrenic or recurrent nerves).

## Limitations

This paper has several limitations. Firstly, the Delphi method has a limitation itself due to to the possibility of a poor response rate. In our study, a high response rate was achieved (82%) because all the selected experts who completed the Delphi round and a high rate of agreement by reaching consensus within the first round. The results also demonstrate the opinions and practice patterns of the content experts with specific expertise in the field of BTS. A second possible limitation is a consensus group on BTS based on individual experience of skilled experts. The consensus is directed at the general thoracic surgical community that face on a daily basis bleeding problems and sometimes the management may also differ based on the surgeon's skills. However, the strength of the Delphi method depends on the participating experts. In the Delphi method, experts' votes were uniformly weighted. The experts were also blinded to the personal opinions of the other participants to reduce peer pressure from influential experts, thereby granted optimal utilization of mutual knowledge and providing access to the change of opinion of the experts. The potential selection bias creating by assembling a group of experts with the same interests, opinions and, in some cases, with different surgical volumes may represents another limitation. Finally, the small sample size of the working group limits the possibility to perform subgroup analysis evaluating differences in responses based on geography and experience. Despite these limitations, the results of this consensus report outlined the importance of recognizing the causes of BTS and to manage the bleeding using appropriate tools aided by the VIBeS, and supporting continued research efforts in this field.

## Conclusions

This expert panel Delphi reached a collective agreement among experts of Italian thoracic surgeons on the majority of issues regarding the factors influencing BTS and the importance of the use of a VIBeS in managing intraoperative bleeding. In addition, according to Delphi survey, a strong agreement was also reached as to the consequences of BTS and as to the role and indications for the The original contributions presented in the study use of appropriate hemostatic agents (active or passive) according to the clinical scenario. In the absence of prospective and randomized studies on management of BTS, this document may be useful for reducing practice variation among thoracic surgeons facing intraoperative bleeding.

## Data Availability

The original contributions presented in the study are included in the article supplementary material, further inquiries can be directed to the corresponding author/s.

## References

[1] Neef H Erbe HJ. Bleeding complications after lung surgery (author’s transl). Zentralbl Chir. (1977) 102:664–9. 899336

[2] Sawada S Komori E Yamashita M. Evaluation of video-assisted thoracoscopic surgery lobectomy requiring emergency conversion to thoracotomy. Eur J Cardiothorac Surg. (2009) 36:487–90. 10.1016/j.ejcts.2009.04.004 19502073

[3] Rostad H Strand TE Naalsund A Talleraas O Norsteon J. Lung cancer surgery: the first 60 days. A population based study. Eur J Cardiothorac Surg. (2006) 29:824–8. 10.1016/j.ejcts.2006.01.055 16520052

[4] Peterffy A Henze A. Hemorragic complications during pulmonary resection. A retrospective review of 1428 resection with 113 hemorragic episodes. Scand J Thorac Cardiovasc Surg. (1983) 17:283–7. 10.3109/14017438309099366 6359394

[5] Sirbu H Busch T Aleksic I Lotfi S Ruschewski W Dalichau H. Chest re-exploration for complications after lung surgery. Thorac Cardiovasc Surg. (1999) 47:73–6. 10.1055/s-2007-1013114 10363604

[6] Szwerc MF Landreneau RJ Santos RS Keenan RJ Murray GF. Mini-toracotomy combined with mechanical stapled bronchial and vascular ligation for anatomical lung resection. Ann Thorac Surg. (2004) 77:1904–10. 10.1016/j.athoracsur.2003.12.003 15172234

[7] Hensen HJ Petersen RH Christensen M. Video-assisted thoracoscopic surgery (VATS) lobectomy using a standardized anterior approach. Surg Endosc. (2011) 25:1263–9. 10.1007/s00464-010-1355-9 20927543

[8] Krasna MJ Deshmukh S McLaughlin JC. Complications of thoracoscopy. Ann Thorac Surg. (1996) 61:1066–9. 10.1016/0003-4975(96)00021-5 8607657

[9] Solaini L Prusciano F Bagioni P Di Francesco F Basilio Poddie D. Video-assisted thoracic surgery major pulmonary resections. Present experience. Eur J Cardiothorac Surg. (2001) 20:437–42. 10.1016/s1010-7940(01)00850-8 11509260

[10] Foroulis CN Kleontas A Karatzopoulos A Nana C Takaragkis G Tossios P Early reoperation performed for the management of complications in patients undergoing general thoracic surgical procedures. J Thorac Dis. (2014) 6(Suppl 1):S21–31. 10.3978/j.issn.2072-1439.2014.02.22 24672696 PMC3966157

[11] Yang Y Gao W Zhao H Yang Y Shi J Sun Y Risk factors and consequences of perioperative reoperation in patients undergoing pulmonary resection surgery. Surgery. (2016) 159:591–601. 10.1016/j.surg.2015.07.030 26365946

[12] Pass SE Simpson RW. Discontinuation and reinstitution of medications during the perioperative period. Am J Health Syst Pharm. (2004) 61:899–912. 10.1093/ajhp/61.9.899 15156966

[13] Doddoli C Thomas P Thirion X Ghez O Payan-Defais MJ Guidicelli R Postoperative complications in relation with induction therapy for lung cancer. Eur J Cardiothorac Surg. (2001) 20:385–90. 10.1016/s1010-7940(01)00764-3 11463562

[14] DeAnda A Elefteriades J Hasaniya N Lattouf OM Lazzara RR. Improving outcomes through the use of surgical sealants for anastomotic sealing during cardiovascular surgery. J Card Surg. (2009) 24(3):325–33. 10.1111/j.1540-8191.2009.00809.x 19438792

[15] Samudrala S. Topical hemostatic agents in surgery: a surgeon’s perspective. AORN J. (2008) 88(3):S2–11. 10.1016/S0001-2092(08)00586-3 18790097

[16] Lewis K Li Q Jones D Correlas J Du H Spiess PE Development and validation of an intraoperative bleeding severity scale for use in clinical studies of hemostatic agents. Surgery. (2017) 161(3):771–81. 10.1016/j.surg.2016.09.022 27839931

[17] VIBe Scale. Baxter Specialty Site (2023). Available online at: https://advancedsurgery.baxter.com/vibe (Accessed April 16, 2023).

[18] DeAnda A Lehman R Lewis K Lo Menzo E Sciubba D Shander A Clinical utility and relevance of a validated intraoperative bleeding scale (VIBe SCALE). J Am Coll Surg. (2019) 229(4):e20. 10.1016/j.jamcollsurg.2019.08.841

[19] Ramirez M Ramirez Almazán M Lewis K. Psu6 economic impact of a validated intraoperative bleeding scale: a retrospective multicenter review from the US prospective. Value Health. (2019) 22:S893. 10.1016/j.jval.2019.09.2593

[20] Graham B Regehr G Wright JG. Delphi as a method to establish consensus for diagnostic criteria. J Clin Epidemiol. (2003) 56:1150e6. 10.1016/s0895-4356(03)00211-7 14680664

[21] Jones J Hunter D. Consensus method for medical and health services research. Br Med J. (1995) 311:376–80. 10.1136/bmj.311.7001.376 7640549 PMC2550437

[22] Yan TD Cao C D'Amico TA Demmy TL He J Hansen H Video-assisted thoracoscopic surgery lobectomy at 20 years: a consensus statement. Eur J Cardiothorac Surg. (2014) 45:633–9. 10.1093/ejcts/ezt463 24130372

[23] Fink A Kosecoff J Chassin M Brook RH. Consensus methods: characteristics and guidelines for use. Am J Public Health. (1984) 74:979–83. 10.2105/ajph.74.9.979 6380323 PMC1651783

[24] Cardillo G Nosotti M Scarci M Torre M Alloisio M Benvenuti MR Air leak and intraoperative bleeding in thoracic surgery: a Delphi consensus among the members of Italian society of thoracic surgery. J Thorac Dis. (2022) 14(10):3842–53. 10.21037/jtd-22-619 36389328 PMC9641338

[25] Bruttomesso D Laviola L Avogaro A Bonora E Del Ptrato S Frontoni S The use of real time continuous glucose monitoring or flash glucose monitoring in the management of diabetes: a consensus view of Italian diabetes experts using the Delphi method. Nutr Metab Cardiovasc Dis. (2019) 29(5):421–31. 10.1016/j.numecd.2019.01.018 30952574

[26] Raison N Wood T Brunckhorst O Abe T Ross T Challacombe B Development and validation of a tool for non-technical skills evaluation in robotic surgery-the ICARS system. Surg Endosc. (2017) 31:5403–10. 10.1007/s00464-017-5622-x 28634630

[27] Camaschella C. Iron-deficiency anemia. N Engl J Med. (2015) 372(19):1832–43. 10.1056/NEJMra1401038 25946282

[28] Mueller M van Remoortel H Meybohm P Aranko K Aubron C Burger R Patient blood management: rRcommendations from the 2018 Frankfurt consensus conference. JAMA. (2019) 321(10):983–7. 10.1001/jama.20190554 30860564

