# Supplementary material for: A Delphi consensus report of the Italian society of thoracic surgery on influencing factors, use of a bleeding scale, and management of bleeding in thoracic surgery
Source: Front Surg. 2026 Apr 28;13:1734148. doi: 10.3389/fsurg.2026.1734148 (PMC13161095; doi:10.3389/fsurg.2026.1734148)
Supplement: Supplementary file 1 [file Supplementaryfile1.docx]

**Appendix**

***Collaborators and Participating Institutions***

Agneta Giuseppe, Division of Thoracic Surgery, Villa Sofia- Cervello Hospital, Palermo, Italy; Ambrogi Vincenzo, Division of Thoracic Surgery, Tor vergata University, Rome, Italy; Aramini Beatrice, Division of Thoracic Surgery, University of Modena, Italy; Astaneh Arash; Division of Thoracic Surgery, Niguarda Hospital, Milan, Italy; Benvenuti Mauro Roberto, Division of Thoracic Surgery, Spedali Civili Hospital, Brescia, Italy; Bertani Alessandro, Division of Thoracic Surgery and Lung Transplantation, Department for the Treatment and Study of Cardiothoracic Diseases and Cardiothoracic Transplantation, IRCCS ISMETT-UPMC, Palermo, Italy; Bottoni Edoardo, Division of Thoracic Surgery, IRCCS Humanitas, Rozzano, Italy; Breda Cristiano, Division of Thoracic Surgery, Ospedale dell'Angelo, Mestre, Italy; Cagini Lucio, University of Perugia, Thoracic Surgery Unit Ospedale del Mare, Naples, Italy; Campisi Alessio, Department of Thoracic Surgery, University and Hospital Trust-Ospedale Borgo Trento, Verona, Italy; Casablanca Giuseppe, Department of Thoracic Surgery, SC AO Papardo of Messina, Messina, Italy; Casaccia Marco, Division of Thoracic Surgery, Pescara Hospital, Pescara, Italy; Cattoni Maria, Division of Thoracic Surgery, Ospedale di Circolo, Varese, Italy; D'Andrilli Antonio, Division of Thoracic Surgery, S. Andrea Hospital, Roma, Italy; Divisi Duilio, Department of Life, Health and Environmental Sciences, Thoracic Surgery Unit, University of L'Aquila, L'Aquila, Italy; Dolci Giampiero, Division of Thoracic Surgery, S. Orsola Hospital, Bologna, Italy; Donati Giovanni, Division of Thoracic Surgery, Aosta Hospital, Aosta, Italy; Ferrari Paolo Albino, Division of Thoracic Surgery, Brotzu Hospital, Cagliari, Italy; Filosso Pier Luigi, Division of Thoracic Surgery, University of Modena, Italy; Fiorelli Alfonso, Division of Thoracic Surgery, Luigi Vanvitelli University, Naples, Italy; Gavezzoli Diego, Division of Thoracic Surgery, Ospedale S. Cuore Don Calabria, Negrar, Italy; Guerrera Francesco, Department of Thoracic Surgery, Azienda Ospedaliera Universitaria Città della Salute e della Scienza di Torino, Torino, Italy; Jaus Massimo, Division of Thoracic Surgery, Forlanini Hospital, Roma, Italy; Leo Francesco, Division of Thoracic Surgery, San Luigi Gonzaga Hospital, Orbassano, Italy; Leuzzi Giovanni, Division of Thoracic Surgery, Fondazione IRCCS Istituto Nazionale dei Tumori, Milan, Italy; Loizzi Domenico, Division of Thoracic Surgery, University of Foggia, Italy; Lomangino Ivan, Division of Thoracic Surgery, Spedali Civili di Brescia, Brescia, Italy; Lopez Camillo, Division of Thoracic Surgery, Vito Fazzi Hospital, Lecce, Italy; Luzzi Luca, Division of Thoracic Surgery, University Hospital, Siena, Italy; Macri' Paolo, Division of Thoracic Surgery, Istituto Clinico Catanese, Catania, Italy; Marulli Giuseppe, Unit of Thoracic Surgery, University of Bari "Aldo Moro", Bari, Italy; Meacci Elisa, Department of General Thoracic Surgery, Fondazione Policlinico Universitario A. Gemelli IRCCS, Università Cattolica del Sacro Cuore, Rome, Italy; Melloni Giulio, Division of Thoracic Surgery, San Matteo Hospital, Pavia, Italy; Mendogni Paolo, Division of Thoracic Surgery, Policlico University Hospital, Milan, Italy; Mercadante Edoardo, Division of Thoracic Surgery, IRCCS Pascale, Naples, Italy; Migliore Marcello, Department Thoracic Surgery, Dept of General Surgery and Medical Specialities and Minimally Invasive Thoracic Surgery and New Technologies, Policlinico University Hospital, University of Catania, Catania, Italy; Monaco Francesco, Division of Thoracic Surgery, University of Messina, Messina, Italy; Nicolosi Tommaso, Division of Thoracic Surgery, Policlinico Morgagni, Catania, Italy; Novellis Pierluigi, Department of Thoracic Surgery, Vita-Salute San Raffaele University, Milan, Italy; Paci Massimiliano, Division of Thoracic Surgery, University of Reggio Emilia, Reggio Emilia, Italy; Pariscenti Gian Luca, Division of Thoracic Surgery, S. Martino Hospital, Genova, Italy; Potenza Enrico, Division of Thoracic Surgery, Garibaldi Hospital, Catania, Italy; Puma Francesco, Division of Thoracic Surgery, University Hospital, Perugia, Italy; Ragusa Mark, Division of Thoracic Surgery, University Hospital, Perugia, Italy; Sollitto Francesco, Division of Thoracic Surgery, University of Foggia, Italy; Tamburini Nicola, Division of Thoracic Surgery, University of Ferrara, Italy; Taurchini Marco, Division of Thoracic Surgery, Division of Thoracic Surgery, Taranto, Italy; Voci Carlopietro, Magna Grecia University, Catanzaro, Italy; Zaraca Francesco, Department of Vascular and Thoracic Surgery, Regional Hospital, Bozen/Bolzano, Italy.
